# Supplementary figures and images for: Semaphorin 4B is an ADAM17-cleaved adipokine that inhibits adipocyte differentiation and thermogenesis
Source: Mol Metab. 2023 Apr 28;73:101731. doi: 10.1016/j.molmet.2023.101731 (PMC10197113; doi:10.1016/j.molmet.2023.101731)

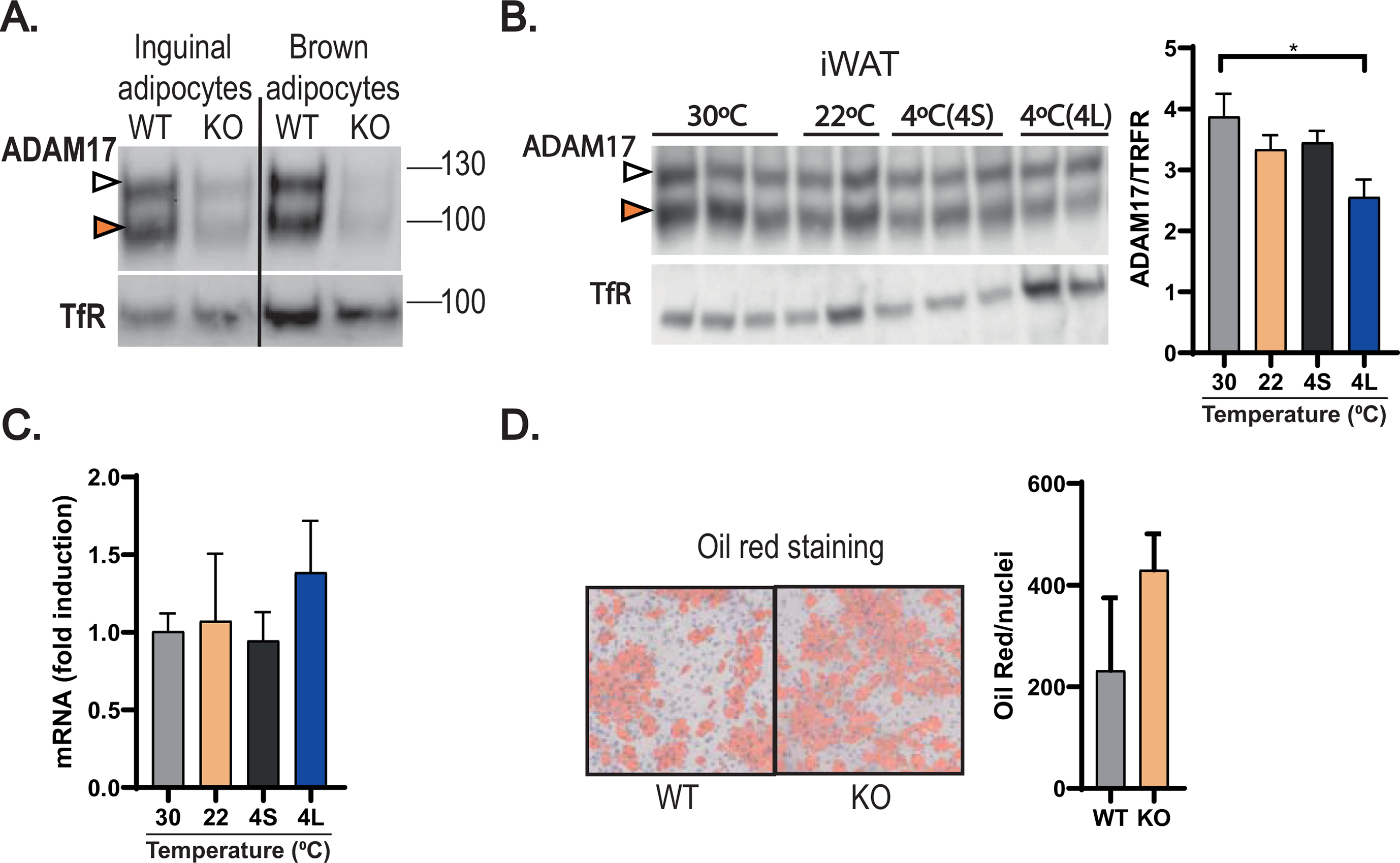

Supplement: Supplementary Figure 1 — Changes in ambient temperature have no impact on inguinal adipose tissue ADAM17. (A), Immunoblot of ADAM17 in adipocytes differentiated from WT and KO inguinal WAT and interscapular BAT. (B), Immunoblot showing mature (100 KDa) (orange arrowhead) and immature (130 KDa) (white arrowhead) ADAM17 levels in inguinal WAT from WT mice exposed to different ambient temperatures; thermoneutrality (30 °C), room temperature (22 °C), acute cold exposure (4° S) (4 °C for 6 h), and chronic cold exposure (4° L) (4 °C for 10 days). Total ADAM17 (mature and immature ADAM17) quantified as a ratio of the loading control, transferrin receptor (TfR) (n = 6 all conditions except n = 4 for 22 °C). (C), mRNA level of Adam17 in the inguinal WAT of mice from B (n = 6). (D), Oil red staining image of differentiated WT and ADAM17 KO primary inguinal adipocytes and oil red quantification per nucleus of the respective images. Results presented as mean ± SD. ∗P < 0.05. [file figs1.jpg]

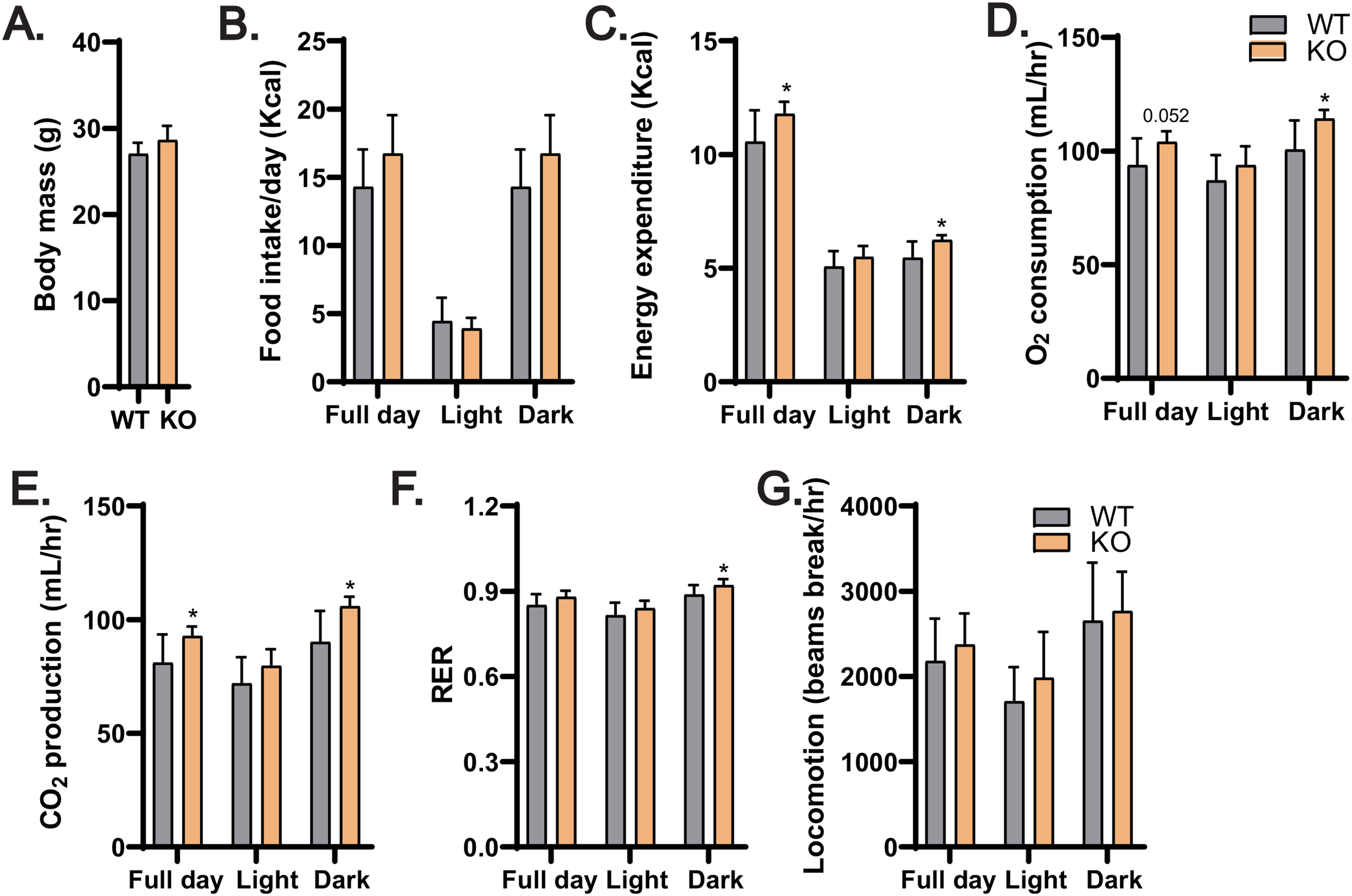

Supplement: Supplementary Figure 2 — Mice null for ADAM17 in adipose tissue have enhanced energy expenditure on standard chow diet. (A-B) Body mass (A) and 24 h food intake (B) of lean WT and KO mice (WT n = 8, KO n = 7). (C–F) Energy expenditure (C), oxygen consumption (D), carbon dioxide production (E), and respiratory exchange ratio (F) of lean WT and KO mice (WT n = 8, KO n = 7). (G), Locomotion of lean WT and KO mice (WT n = 8, KO n = 7). Results presented as mean ± SD. ∗P < 0.05. [file figs2.jpg]

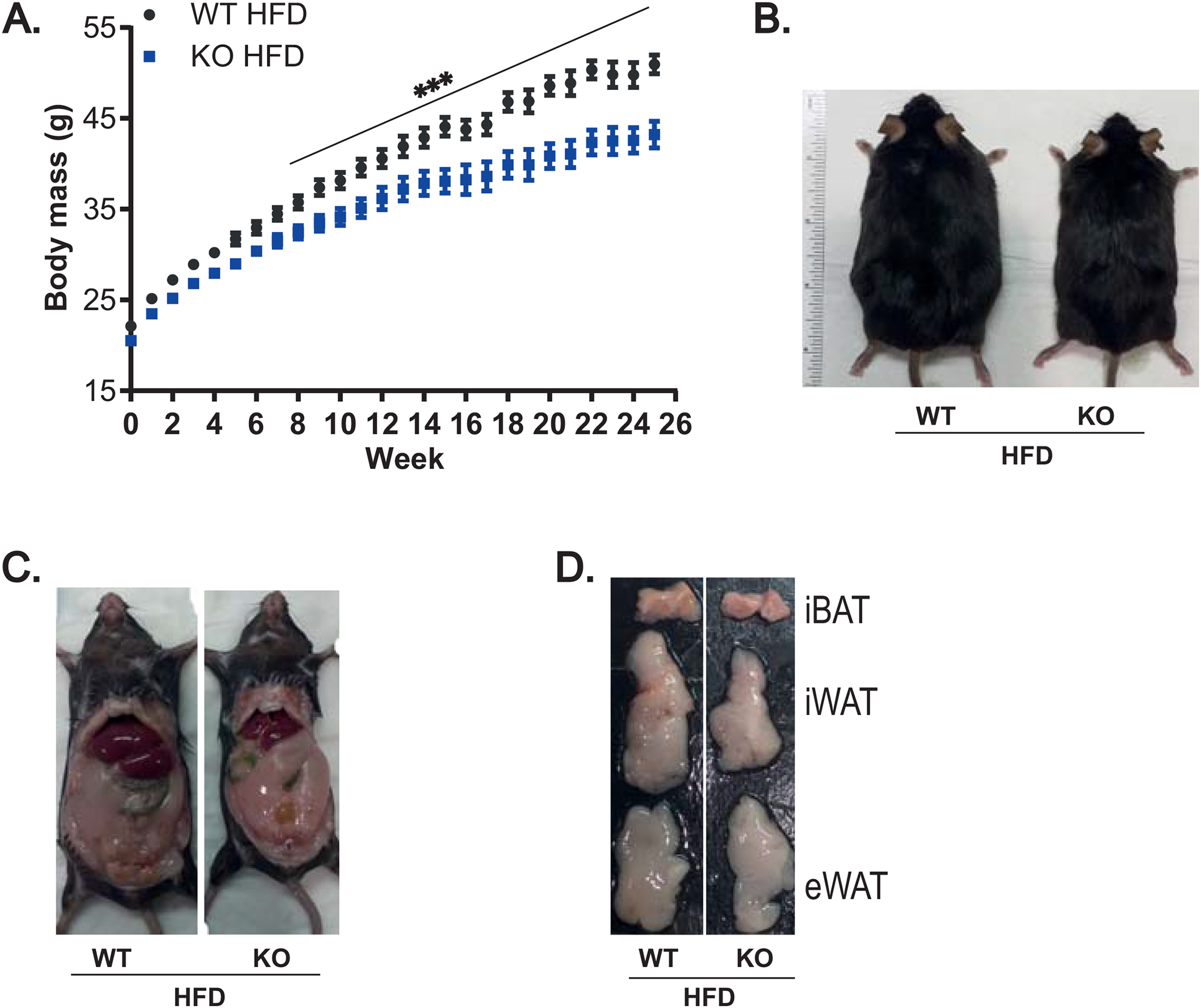

Supplement: Supplementary Figure 3 — Mice null for ADAM17 in adipose tissue are less obese upon HFD feeding. (A), Change in body weight in WT and KO mice over the 26-week period of HFD feeding (WT n = 20, KO n = 18). (B–D), Images of obese (B) WT and KO mice, of their abdominal organs (C), and of their interscapular BAT, inguinal WAT, and epididymal WAT (D) after 26 weeks of HFD feeding. Results presented as mean ± SD. ∗P < 0.05, ∗∗P < 0.01, ∗∗∗P < 0.001, ∗∗∗∗P < 0.0001. [file figs3.jpg]

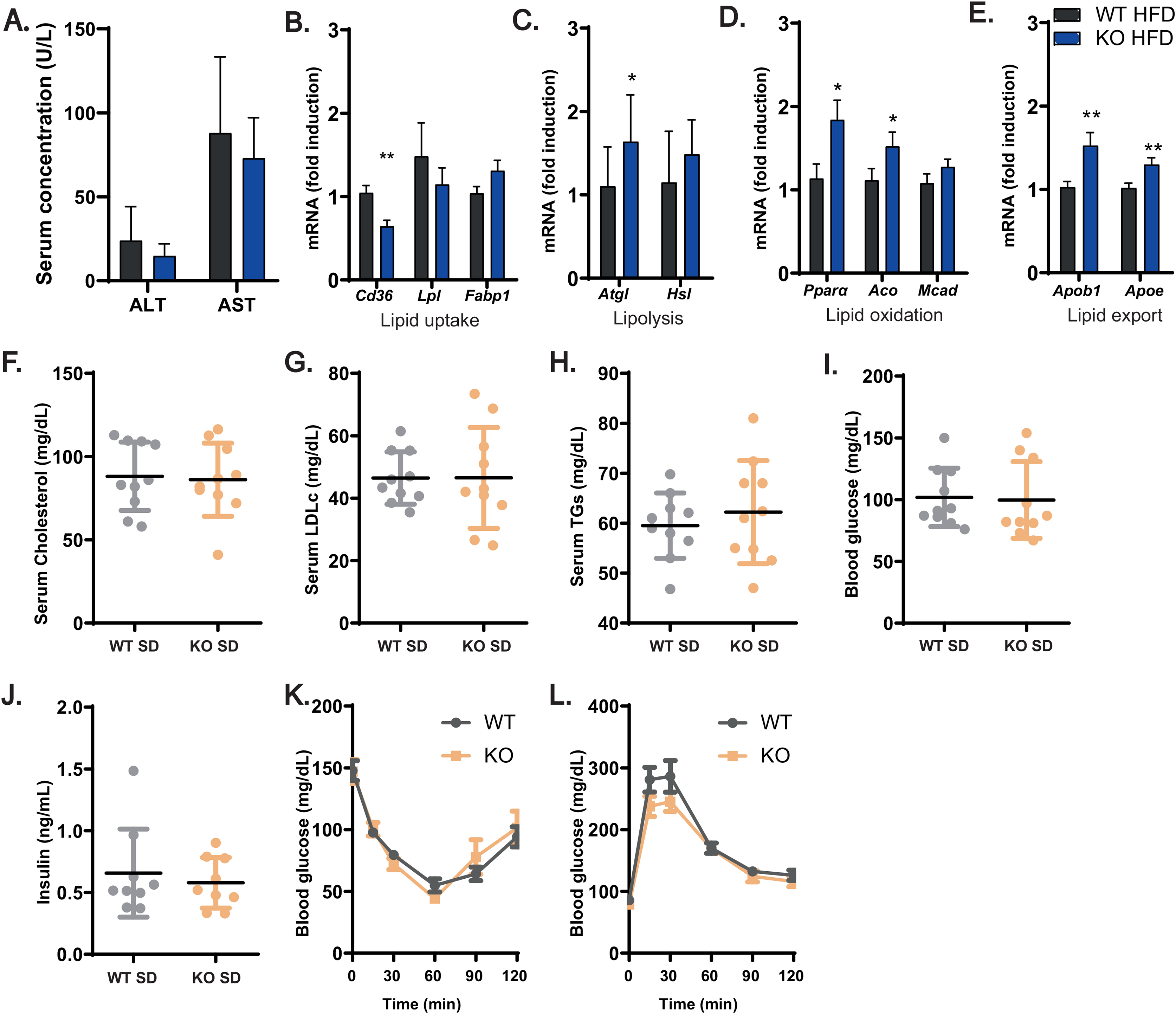

Supplement: Supplementary Figure 4 — Loss of ADAM17 in adipocytes promotes lipid hepatic lipid catabolism in obesity but has no metabolic impact in lean condition. (A) ALT and AST quantification in the livers of obese WT and KO mice (n = 5).(B–E), mRNA expression of genes involved in lipid uptake (B), lipolysis (C), lipid oxidation (D), and lipid export (E) in the livers of obese WT and KO mice (WT n = 13, KO n = 12). (F–H), Fasting serum levels of total cholesterol (F), LDLc (G), and triglycerides (TGs) (H) in lean WT and KO mice (n = 10). (I–J), Fasting blood glucose (I) and serum insulin levels (J) of lean WT and KO mice (n = 10). (K–L), Insulin (K) and glucose (L) tolerance tests in lean WT and KO mice (n = 10). Results presented as mean ± SD. ∗P < 0.05, ∗∗P < 0.01, ∗∗∗P < 0.001, ∗∗∗∗P < 0.0001. [file figs4.jpg]

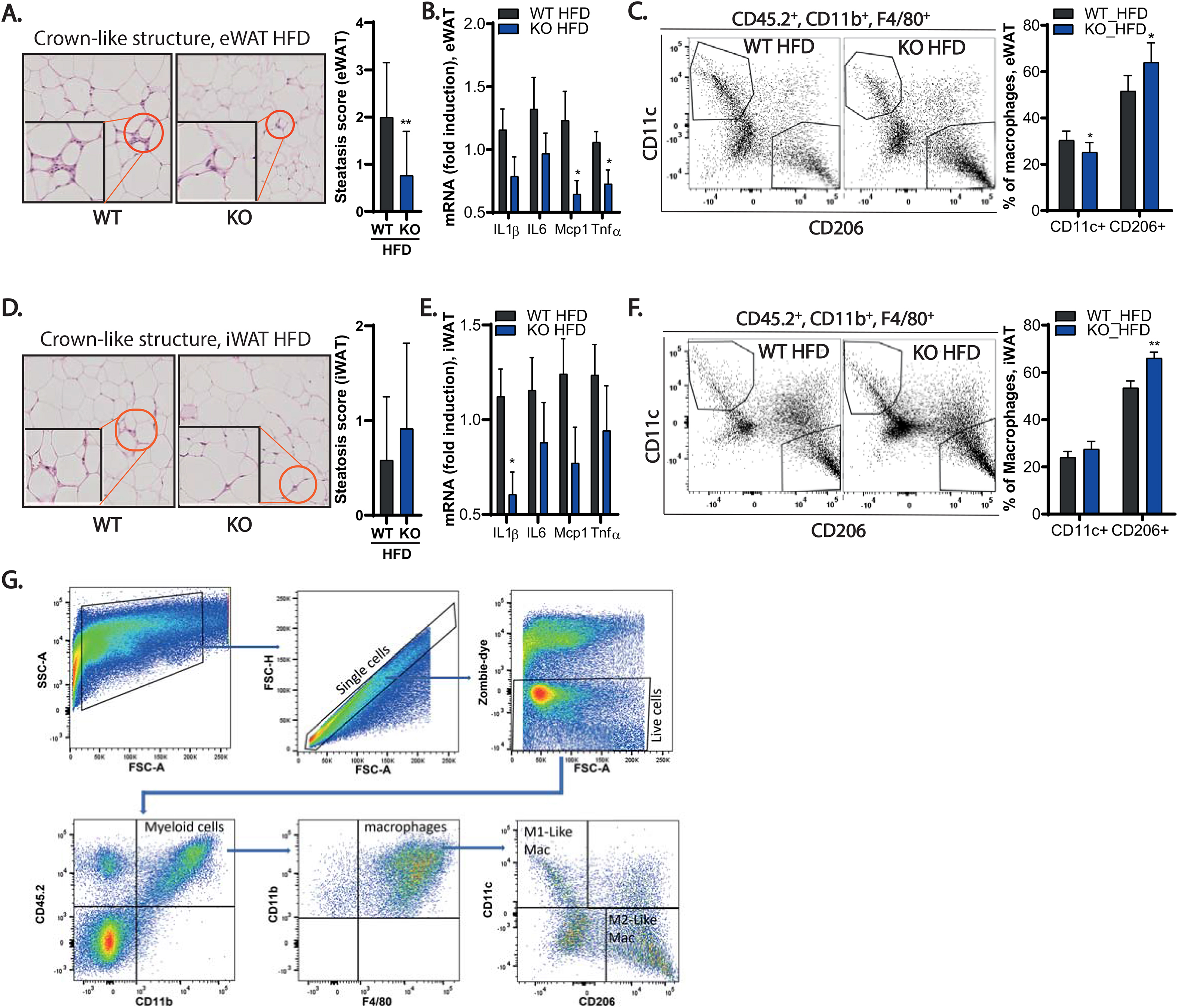

Supplement: Supplementary Figure 5 — Loss of ADAM17 in adipocytes dampens adipose tissue inflammation in obesity. (A), H&E staining epididymal WAT (eWAT) slide from obese WT and KO mice and quantification of adipose tissue steatitis (inflammation). (B), mRNA expression of proinflammatory genes; Il1β, Il6, Mcp1, and Tnfα in epididymal WAT of obese WT and KO mice. (C), Flow cytometry analysis of the polarity (M1, M2) of macrophages in eWAT of obese WT and KO mice. (D), H&E staining inguinal WAT (iWAT) slide from obese WT and KO mice and quantification of steatitis (inflammation). (E), mRNA expression of proinflammatory genes; Il1β, Il6, Mcp1, and Tnfα in iWAT of obese WT and KO mice. (F), Flow cytometry analysis of the polarity (M1, M2) of macrophages in iWAT of obese WT and KO mice. (G), Gating strategy used in the flow cytometry analysis of macrophage population in the eWAT and iWAT of obese WT and KO mice (n = 7). Results presented as mean ± SD. ∗P < 0.05, ∗∗P < 0.01. [file figs5.jpg]

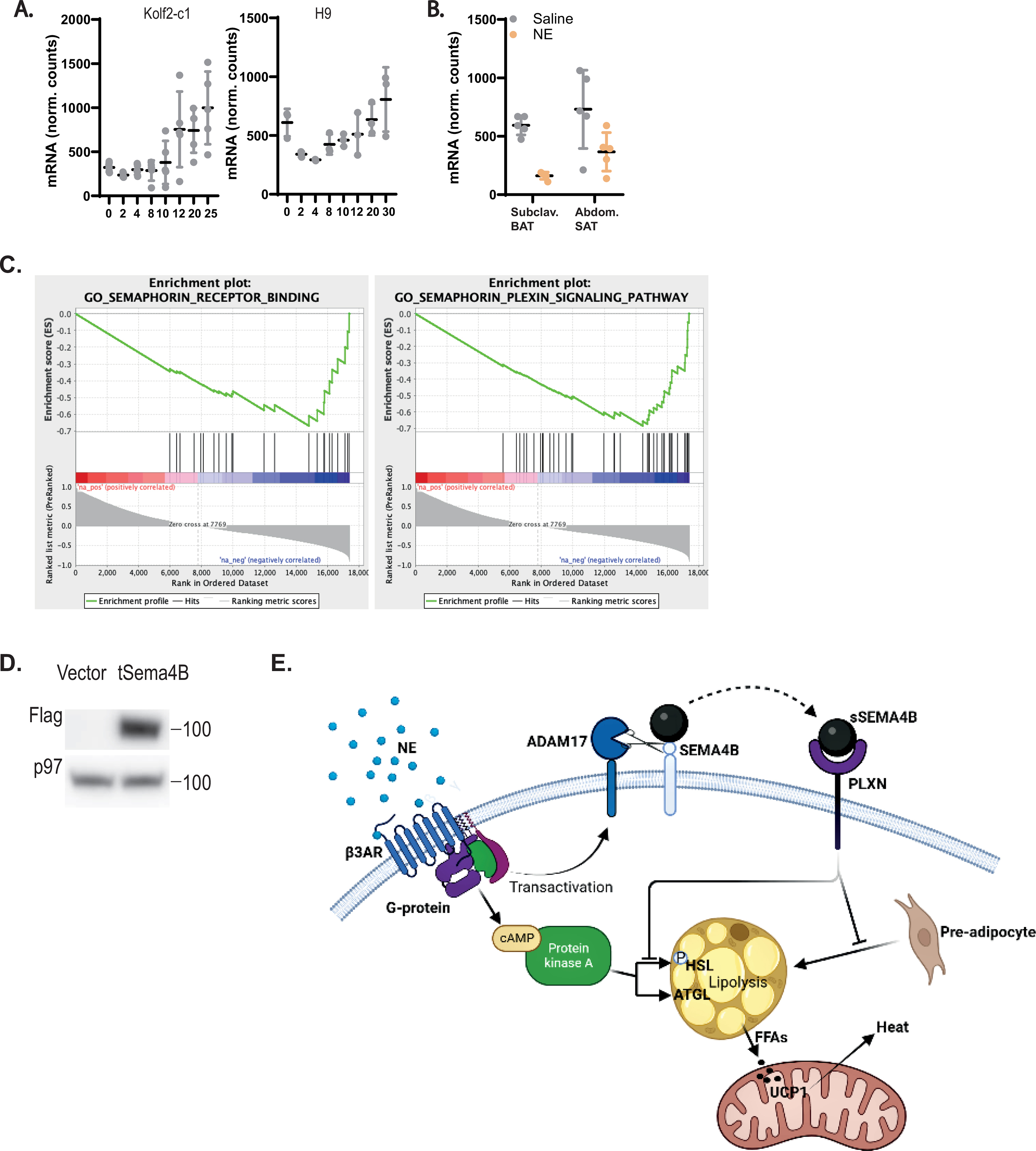

Supplement: Supplementary Figure 6 — Semaphorin signaling inversely correlates with UCP1 expression (A), mRNA expression of Sema4B during differentiation of human stem cells Kolf2-c1 and H9 into matured brown adipocytes ex vivo. (B), mRNA expression of Sema4B in adipocytes differentiated from human subclavicular BAT and abdominal SAT with or without stimulation with norepinephrine (NE). (C), Human gene set enrichment analysis (GSEA) from RNA seq data from Din et al. Genes enrich for the GO:TERM “semaphorin receptor binding” and “semaphorin plexin signaling pathway” negatively correlate with UCP1 mRNA expression. (D), Anti-FLAG immunoblot on lysates of immortalized primary brown adipocytes transduced with empty vector or Flag-tagged truncated Sema4B (tSema4B). (E) Proposed schematic for how ADAM17-cleaved Sema4B negatively regulates adipocyte differentiation and lipolysis which in turn impact on substrate (fatty acid) availability for activation of UCP1 for thermogenesis. In parallel to the canonical beta-adrenergic pathway that drives lipolysis to support thermogenesis, our data show that beta-adrenergic receptor activation triggers ADAM17 activation and the cleavage of Sema4B, to inhibit adipogenesis and thermogenesis. Results presented as mean ± SD. [file figs6.jpg]
